# Roles of cytotoxic lymphocytes and MIC/LILR families in pathophysiology of Takayasu arteritis

**DOI:** 10.1186/s41232-020-00119-6

**Published:** 2020-06-02

**Authors:** Hajime Yoshifuji, Chikashi Terao

**Affiliations:** 1 Department of Rheumatology and Clinical Immunology, Graduate School of Medicine, Kyoto University, 54 Shogoin Kawahara-cho, Sakyo-ku, Kyoto, 606-8507 Japan; 2 Laboratory for Statistical and Translational Genetics, RIKEN Center for Integrative Medical Sciences, Kanagawa, Japan; 3 Clinical Research Center, Shizuoka General Hospital, Shizuoka, Japan; 4 The Department of Applied Genetics, The School of Pharmaceutical Sciences, University of Shizuoka, Shizuoka, Japan

**Keywords:** Vasculitis, Genome-wide association study, Natural killer cell, Cytotoxic T cell, Interleukin-6, Interleukin-12, MICA

## Abstract

Takayasu arteritis (TAK) affects the aorta and its primary branches, mainly in young women. In its advanced stages, it can cause severe complications, such as cerebral infarction, impaired vision, and valvular heart diseases. In the aortic tissue of TAK, there is increased infiltration of cytotoxic lymphocytes, such as natural killer (NK) cells and CD8^+^T cells, and enhanced expression of accessory molecules, such as major histocompatibility complex (MHC) and MHC class I chain-related gene (MIC) family. Genome-wide association studies on TAK have identified susceptibility genes, such as IL-12p40, MICA, MICB, leukocyte immunoglobulin-like receptor A3 (LILRA3), and LILRB3. Other studies have also shown their involvement in the pathophysiology of TAK. In addition, we reported the importance of NK cells by enhancer enrichment analysis. These results suggest that the gene polymorphisms that potentially upregulate the expression of cytokines and accessory molecules, which contribute to the activation of cytotoxic lymphocytes, are associated with the development of TAK. Based on these results, new molecular targeted therapies look promising.

## Background

Takayasu arteritis (TAK) is a rare disease belonging to the vasculitis syndrome that frequently occurs in young women [1]. Although glucocorticoids and immunosuppressants are standard treatments, the disease often relapses. In its advanced stages, serious complications such as cerebral ischemia, visual loss, and aortic aneurysms may occur. Elucidation of disease mechanisms is needed to develop new effective treatments.

The physiological role of cytotoxic lymphocytes, such as CD8^+^T cells and natural killer (NK) cells, is to eliminate the cells affected by malignancy or intracellular microorganisms. They are regulated by accessory molecules, such as major histocompatibility complex (MHC), MHC class I chain-related gene (MIC) family, and leukocyte immunoglobulin-like receptor (LILR) family. In this review, the roles of cytotoxic lymphocytes and accessory molecules in the pathogenesis of TAK are discussed.

## Main text

### Pathophysiology of TAK

**Fig. 1:**
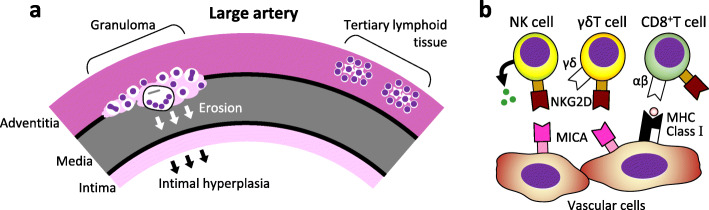
**a** A pathological schema of the aorta affected by TAK. **b** Pathophysiology of aortic lesions

In the pathophysiology of TAK, the aorta and its primary branches are inflamed, causing stenosis of the arterial lumen, which leads to ischemia of downstream organs [1, 2]. Occasionally, the arterial lumen is dilated, causing aortic regurgitation and aneurysms. Histologically, the layers of an arterial wall consist of the adventitia, media, and intima (Fig. 1a) [2]. In particular, large arteries have thick media with rich elastic fibers. In the early stage of TAK, inflammatory cells infiltrate the adventitia [2]. Gradually, granulomas are formed adjacent to the media with monocytes, lymphocytes, neutrophils, epithelioid cells, and giant cells [3], causing the erosion of the media from the outside. In response to medial destruction, intimal thickening occurs and leads to stenosis of the arterial lumen.

In the aortic tissue of TAK (Fig. 1b), Seko et al. [4, 5] reported increased infiltration of NK cells, γδT cells, and CD8^+^T cells, and enhanced expression of accessory molecules, such as MHC, MICA, and natural killer group 2 member D (NKG2D), and suggested their involvement in the pathophysiology of TAK.

Genome-wide association studies (GWAS) have been conducted to search for genes susceptible to TAK [6–9] (Table 1). *Human leukocyte antigen (HLA)-B* has been shown to have the strongest association. This is consistent with the classically reported association of HLA-B52 with TAK [10]. Because HLA-B52 belongs to MHC class I, the results support the involvement of CD8^+^T cells in the pathophysiology of TAK. Nevertheless, there remains a possibility that the association of *HLA-B* is not authentic, as described below.

**Table 1:** Gene regions associated with TAK

Author, year	Gene region
Terao, 2013 [6]	*IL12B*, *HLA-B*, *MLX*
Saruhan-Direskeneli, 2013 [7]	*FCGR2A/FCGR3A*, *IL12B*, *HLA-B/MICA*, *HLA-DQB1/HLA-DRB1*
Renauer, 2015 [8]	*HLA-B/MICA*, *IL6*, *RPS9/LILRB3*, *chr21q22*
Terao, 2018 [9]	*FCGR3A*, *IL12B*, *DUSP22*, *HLA-B*, *PTK2B*, *KLHL33*, *LILRA3*, *chr21q22*

### Involvement of IL-12p40

**Fig. 2:**
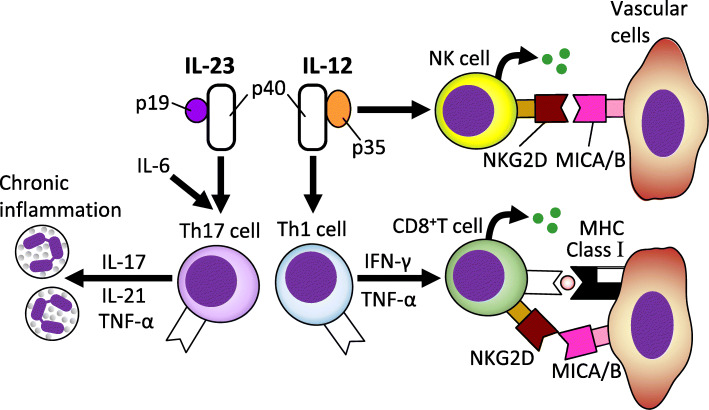
Cytokines in the pathophysiology of TAK

Single nucleotide polymorphisms (SNPs) in the *IL12B* gene region have been found in GWAS using two separate cohorts [6, 7]. Hence, the involvement of this genetic region in the pathogenesis of TAK is highly probable. *IL12B* encodes p40, a subunit shared by IL-12 and IL-23 (Fig. 2) [2]. IL-12 and IL-23 are essential factors for the differentiation and maintenance of T helper-1 (Th1) and T helper-17 (Th17) cells, respectively. Th1 can activate cytotoxic lymphocytes, suggesting the contribution of these SNPs to the development of TAK.

Classically, genetic mutations have been located in the coding regions of the genome and affect the structure of proteins. However, most SNPs found by GWAS are located in the non-coding regions and probably affect the expression level and splicing of mRNA [11]. Genetic factors that affect the expression levels of mRNA are called “expression quantitative trait loci (eQTL).” We hypothesized that the *IL12B* SNP (rs6871626) located in a non-coding region has an eQTL effect on *IL12B* gene expression. We showed that p40 is more highly expressed in patients with the risk allele of the SNP [12]. Moreover, the risk allele was significantly associated with patients’ clinical features, such as complication of aortic regurgitation [6], inflammatory marker levels [6], and refractory courses [13]. Hence, p40 appears to be pivotal in the pathophysiology of TAK. We performed a pilot study using anti-p40 monoclonal antibodies (ustekinumab) for three refractory TAK patients who showed improvement of symptoms and decrease in inflammatory markers [14].

### Enhancer enrichment analysis

**Fig. 3:**
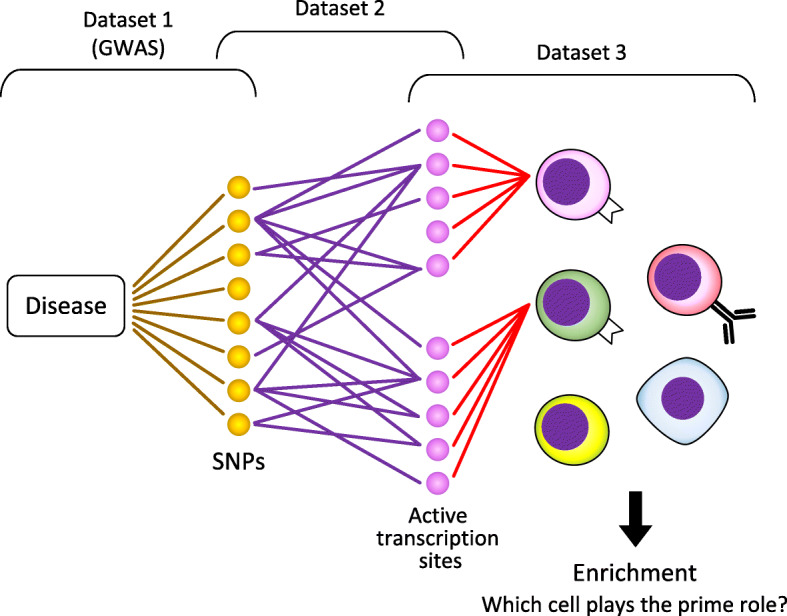
Enhancer enrichment analysis searches for cell types that have “enriched” transcription sites genetically linked to a certain disease. Dataset 1: Associations between a disease and SNPs (GWAS). Dataset 2: Overlap between transcription sites. Dataset 3: Active transcription sites in each cell type

Two study groups have performed enhancer enrichment analyses to examine which cell types play important roles in the pathophysiology of TAK (Table 2) [9, 15]. This method searches for cell types that have “enriched” transcription sites genetically linked to a certain disease, using several open datasets (Fig. 3). A comparison of results showed that NK cells were the highest in our study and the third in the study by Sawalha et al., suggesting the importance of NK cells in the pathophysiology of TAK. Nevertheless, B cells were also at a high position in both studies.

**Table 2:** Enhancer enrichment analyses of TAK

Cells	*P*
Terao, 2018 [9]	
Natural killer cells	0.000088
T CD8^+^ memory cells	0.00079
T helper naïve cells	0.00096
B cells from cord blood	0.00098
Neutrophils	0.0013
T CD8^+^ naïve cells	0.0013
T cells	0.0021
Mononuclear cells	0.0036
Monocytes	0.0039
T regulatory cells	0.01
Sawalha, 2016 [15]	
B cells from peripheral blood	0.00193
Monocytes from peripheral blood	0.00787
Natural killer cells from peripheral blood	0.00802
T cells from peripheral blood	0.0134
Hematopoietic stem cells G-CSF-mobilized (female)	N.S.
Hematopoietic stem cells G-CSF-mobilized (male)	N.S.
T cells from cord blood	N.S.

All cells are primary cells

### Involvement of LILR/MIC families

**Fig. 4:**
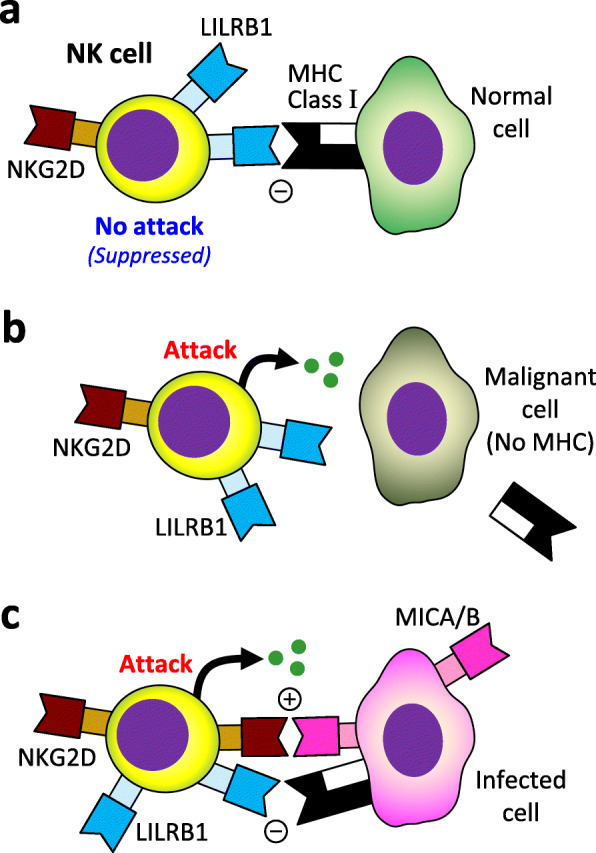
Recognition of normal (**a**), malignant (**b**), and infected cells (**c**) by NK cells

Table 1 includes *LILRA3* and *LILRB3* genes, which belong to the LILR family expressed on various leukocytes. LILRA1 to A6 (except for LILRA3) have immunoreceptor tyrosine-based activation motifs (ITAM) and transmit activation signals into leukocytes, whereas LILRB1 to B5 have immunoreceptor tyrosine-based inhibitory motifs (ITIM) and transmit inhibitory signals. As shown in Fig. 4, it is considered that LILIRB1 recognizes the self MHC, avoiding leukocytes from self-attack. Some malignant cells do not express MHC to escape from CD8^+^T cells, but NK cells can attack them.

Renauer et al. [8] found a SNP in the *RPS9/LILRB3* region and demonstrated its negative eQTL effect on *LILRB3* gene expression. As the SNP has a negative effect on the inhibitory receptor, it might contribute to the development of TAK. Next, we identified SNPs in *LILIRA3* region and found that the lead SNP in *LILIRA3* region tagged the deletion of LILRA3 and demonstrated its strong negative eQTL association with *LILRA3* gene expression [9]. This finding is in contrast to the results of Renauer et al. because it has a negative effect on the activation receptor. However, among most LILRAs that have ITAMs, only LILRA3 lacks its intracellular domain. LILRA3 is considered a secretory molecule and probably a decoy. Thus, our results might not necessarily contradict their results.

The association of HLA-B52 with TAK has been reported since 1978 [10]. However, the SNP discovered by Saruhan-Direskeneli et al. is located in a region between *HLA-B* and *MICA* (Table 1). The MIC family (MICA and MICB) is expressed by the infected cells and recognized by NKG2D on the surface of the killer cells (Fig. 4c) so that the killer cells can attack them. We found a novel SNP in the *MICB* region, which showed a strong linkage disequilibrium with HLA-B52 (*P* = 3.9 × 10^−30^) [9]. Interestingly, the SNP showed a positive eQTL effect on *MICB* gene expression. This suggests that this SNP contributes to the vasculitic condition through the upregulation of MICB on blood vessels and stimulation of killer cells. Moreover, the association of the SNP in the *MICB* region substantially remained after conditioning on HLA-B52 (*P* = 4.0 × 10^−7^), but the association of HLA-B52 was no longer significant after conditioning on the SNP (*P* = 0.17). Considering these findings, the MIC family may be more involved in the pathophysiology of TAK than HLA-B52.

### New therapies for TAK

As described above, our pilot p40 inhibition therapy for TAK showed favorable results. Secondly, IL-6 (its gene is also listed in Table 2) is essential for Th17 induction and plays an important role in inflammatory conditions (Fig. 2). A randomized controlled study using monoclonal antibodies against the IL-6 receptor (tocilizumab) has shown favorable effects for TAK [16]. Additionally, TNF-α is secreted from helper T cells and macrophages and plays an important role in inflammatory conditions (Fig. 2). Many case series have suggested the efficacy of TNF inhibitors for refractory TAK cases [17]. Future detailed studies in these three avenues (p40, IL-6, and TNF) may lead to promising results that may help develop treatment options.

### Significance of autoantibodies

Clement et al. [3] found tertiary lymphoid tissues in the aortic adventitia of TAK (Fig. 1a). These tissues form a germinal center with follicular dendritic cells surrounded by B cells, suggesting the development of antigen-specific B cells. Mutoh et al. [18] found anti-endothelial protein C receptor antibodies and anti-scavenger receptor class B type 1 antibodies in the serum of TAK patients (when combined, sensitivity 67.3% and specificity 98.0%) and showed that they might contribute to the activation of endothelial cells and the promotion of Th17. These findings may be in conflict with our work on cytotoxic immunity. The relationship between autoantibody-related pathophysiology and cytotoxic immunity-related pathogenesis needs to be studied in detail.

## Conclusions

In conclusion, gene polymorphisms that potentially enhance the expression of cytokines and accessory molecules that contribute to the activation of cytotoxic lymphocytes are associated with the development of TAK. There have been an increasing number of studies reporting the efficacies of cytokine inhibition therapies for refractory TAK. These results can lead to the development of new molecular targeted therapies.

## Data Availability

Not applicable.

## Abbreviations

eQTL: Expression quantitative trait loci
GWAS: Genome-wide association study
HLA: Human leukocyte antigen
IFN: Interferon
ITAM: Immunoreceptor tyrosine-based activation motif
ITIM: Immunoreceptor tyrosine-based inhibitory motif
IL: Interleukin
LILR: Leukocyte immunoglobulin-like receptor
MHC: Major histocompatibility complex
MIC: Major histocompatibility complex class I chain-related gene
NK: Natural killer
NKG2D: Natural killer group 2 member D
SNP: Single nucleotide polymorphism
TAK: Takayasu arteritis
Th1: T helper-1
Th17: T helper-17
TNF: Tumor necrosis factor
